# Neonatal indicator data in Tanzania District Health Information System: evaluation of availability and quality of selected newborn indicators, 2015-2022

**DOI:** 10.1186/s12887-025-05417-x

**Published:** 2025-01-23

**Authors:** Josephine Shabani, Nahya Salim, Christine Bohne, Louise Tina Day, Claud Kumalija, Ahmad Mohamed Makuwani, Felix Bundala, Habib Ismail, Joy E. Lawn, Eric O. Ohuma

**Affiliations:** 1 Ifakara Health Institute, Dar es Salaam, Tanzania; 2 Department of Pediatrics and Child Health, Muhimbili University of Health and Allied Sciences (MUHAS), Dar es Salaam, Tanzania; 3 Rice360 Institute for Global Health Technologies, Rice University, Texas, USA; 4 Maternal, Adolescent, Reproductive, & Child Health Centre, London School of Hygiene & Tropical Medicine, London, UK; 5 Ministry of Health, Dodoma, Tanzania

**Keywords:** Newborn, Every newborn action plan, Low- and Middle-Income Countries, District Health Information System, Neonatal mortality rate

## Abstract

**Background:**

The Every Newborn Action Plan (ENAP) indicators are essential in monitoring neonatal healthcare coverage and quality. The District Health Information System (DHIS2), an open-source platform in over 80 countries, supports health data collection and analysis, enabling progress tracking at national and subnational levels. This study evaluates the availability and quality of maternal and newborn health indicators, explicitly focusing on ENAP indicators within Tanzania’s DHIS2.

**Methods:**

Using the EN-MINI tool, we assessed data availability for 20 ENAP indicators by analysing their numerators and denominators in Tanzania’s DHIS2 (2015–2022) across all healthcare levels. World Health Organization’s (WHO) data quality framework was adapted to examine four dimensions: (a) availability of indicators, (b) completeness of indicator reporting, (c) internal consistency of related indicators, and (d) indicator plausibility by comparing DHIS2 data with population-based Demographic and Health Survey (DHS) data.

**Results:**

Of the 20 ENAP indicators, 14 were available in Tanzania’s DHIS2, with definitions, numerators and denominators aligned with WHO standards. Between 2015 and 2022, the number of facilities reporting at least one delivery annually increased by 19% from 5,898 to 7,016. During this period, 75% to 97% of facilities consistently reported data on skilled attendance at birth and early breastfeeding initiation. In contrast, 4% to 54% of facilities reported on maternal and newborn outcomes, including complications such as stillbirths and maternal mortality. Internal consistency was high (> 94%). However, neonatal mortality rates reported in DHIS2 were lower than those reported in Tanzania DHS for similar periods, even after a 20% adjustment to account for home births.

**Conclusion:**

Tanzania’s DHIS2 captures many ENAP indicators; however, notable variability in data quality persists, with substantial data gaps related to maternal and newborn outcomes and complications. To address these challenges, it is crucial to strengthen routine data review, implement robust quality checks, enhance validation processes, provide targeted training, deliver constructive feedback, and conduct supportive supervision. Placing greater emphasis on using DHIS2 data to monitor progress will help identify gaps and drive improvements in data quality, ultimately supporting better maternal and newborn health outcomes.

**Supplementary Information:**

The online version contains supplementary material available at 10.1186/s12887-025-05417-x.

## Key findings

**Table:** 

**What was known?**
• Globally, an estimated 2.3 million newborn deaths and over 1.9 million stillbirths occur annually. Tanzania’s neonatal mortality rate is estimated at 24 per 1000 live births and has not reduced over the last decade. High-burden countries such as Tanzania face challenges accessing reliable neonatal indicator data to monitor progress, guide interventions, and support decision-making. Given that > 80% of births in Tanzania occur in health facilities, routine facility-based data systems like DHIS2 allow data to be captured for most of those receiving care at facilities. • The *Every Newborn* Action Plan (ENAP), launched in 2014, included a list of 20 key indicators for tracking newborn health at the national and global levels. However, the availability and quality of these newborn indicators in Tanzania’s DHIS2 system have not been systematically assessed.
**What was done that is new?**
• Monthly newborn indicator data were extracted from over 7,000 health facilities in the DHIS2 database (2015–2022), encompassing 13 million institutional deliveries. • The EN-MINI Tool was used to map the availability of ENAP indicators and assess their alignment with standard numerator and denominator definitions. • The WHO data quality framework was adapted and applied to standardise ENAP indicator data quality assessments in DHIS2.
**What was found?**
We assessed data quality according to four dimensions: • Indicator availability: ◦ Of the 20 recommended ENAP indicators, 70% (14/20) were available in Tanzania’s DHIS2. Notably, gestational age is - an essential indicator for defining prematurity, a leading risk factor for neonatal mortality is not reported. ◦ The analysis focused on 12 out of 20 available indicators related to intrapartum, maternal, and newborn care. The definitions of these 12 indicators align with standardised WHO ENAP guidelines or nationally recognised DHIS2 core and additional indicator definitions. • Reporting completeness: ◦ < 1% of health facilities in Tanzania reported on all 12/20 available ENAP indicators related to intrapartum, maternal and newborn care. However, > 57% of health facilities reported on at least four available indicators. ◦ There were gaps in reporting of ENAP indicators with considerable variations in reporting that ranged from 4% for treatment of severe neonatal infections indicator to 95.9% for institutional livebirths indicator. Similarly, < 20% of hospitals reported maternal mortality (range: 12–18%) and was worse for health centres (< 5%). Over 50% of hospitals reported stillbirth and neonatal mortality data compared to < 22% of health centres. • Internal consistency and external plausibility: For total stillbirths and total births, > 84% of reported total stillbirths (count of fresh and macerated stillbirths) and total births (count of livebirths and stillbirths) were consistent. For direct comparison between DHIS2 and Tanzania DHS, we adjusted the 2022 DHIS2 estimates for neonatal mortality rate (NMR), maternal mortality rate (MMR) and stillbirth rate (SBR) by 20% to account for home births. The NMR was about 6%, which is implausibly low, underestimating by more than 75% compared to 2022/23 Tanzania DHS. Similarly, the MMR and SBR from DHIS2 were 68 and 10, respectively, reflecting an underestimation of about 50%.
**What next?**
• We propose a thorough evaluation and prioritisation of newborn ENAP indicators for inclusion, focusing on those that align with national and international priorities while addressing existing data gaps. • We recommend the development of a neonatal register for admitted newborns and improvements in the tallying of neonatal deaths in labour wards to strengthen estimates of neonatal mortality. The transition from aggregate data to capturing individual-level data within DHIS2 marks a significant advancement, enhancing the granularity and utility of health information. • We recommend implementing conditional rules to address blank entries in DHIS2 and establishing a clear distinction between actual missing values and entries recorded as ‘zero’.

## Background

The global burden of newborn deaths and stillbirths remains a significant public health concern, with an estimated 2.3 million newborn deaths and 1.9 million stillbirths occurring worldwide in 2022 [1, 2]. The Sustainable Development Goal 3.2 aims to reduce the country’s neonatal mortality rates (NMR) to 12 or less per 1000 live births by 2030. This target was established based on recommendations from the *Every Newborn* Action Plan (ENAP) and the Global Strategy for Women’s, Children’s, and Adolescents’ Health. Similarly, additional targets aim to reduce stillbirth rates to 12 or fewer stillbirths per 1000 total births [3–7].

Sub-Saharan Africa stands out as a region with the highest neonatal mortality, i.e., 45% of the global burden of neonatal deaths [3]. Despite global efforts to reduce NMR, the number of neonatal deaths in sub-Saharan Africa has remained high at around 1 million deaths annually [8–11]. The 2022/23 Tanzania Demographic and Health Survey (TDHS) reported an NMR of 24 per 1000 live births [12], which is not different from the 25 per 1000 live births reported in the 2015/16 TDHS. The proportion of homebirths dropped to 18% in the 2022/23 TDHS [12] from 34% in the 2015/16 TDHS [13]. Most neonatal deaths in Tanzania occur around the time of birth and are attributed to preventable causes such as prematurity, infections, and birth complications. Enhancing World Health Organization (WHO) level 2 special newborn care interventions, including Kangaroo Mother Care (KMC) for all stable neonates < 2000 g, assisted feeding and IV fluids, safe administration of oxygen, detection and management of neonatal sepsis with antibiotics, detection and management of neonatal jaundice with phototherapy are among targeted interventions crucial for reducing neonatal mortality in Tanzania [10, 12, 14–16].

Through various policies and national strategies, Tanzania has demonstrated a strong commitment to Reproductive, Maternal, Newborn, Child, and Adolescent Health (RMNCAH). These include (a) the National Five-Year Development Plan (2021/2022–2025/2026), which aims to enhance the quality of life and well-being for all Tanzanians; (b) the Health Sector Strategic Plan V (2021/22–2025/26) which aims to provide sustainable health services that meet acceptable standards for all citizens, without financial constraints, while ensuring geographical and gender equity [17, 18]and, (c ) the National Plan for Reproductive, Maternal, Newborn, Child and Adolescent Health & Nutrition (2021/2022–2025/2026) which focuses explicitly on improving coverage and quality of health services across the continuum of care (One Plan III).

The country has also made substantial progress in improving coverage and access to institutional delivery rates (currently at > 80%) and births attended by skilled healthcare providers (currently at 85%) [12]. However, challenges remain, especially in the provision of quality care. The lack of quality care is exacerbated by insufficient healthcare providers and patients’ access to Emergency obstetric and newborn care (EmONC) services [19–22]. ENAP encompasses 20 core and additional indicators designed to monitor progress and identify gaps in the care of small and sick newborns (Table 1) [23–26]. The indicators are typically related to various aspects of newborn health and care, such as neonatal mortality rates, coverage of essential interventions, access to skilled care during childbirth, and the quality of maternal and newborn health services [11, 14].

**Table 1:** Core and additional indicators to track impact, coverage for ENAP

Current Status	Level	Core Indicators	Additional indicators
Definitions clear but quantity & consistency of data are lacking	IMPACT	1. Maternal mortality ratio	
		**2. Stillbirth rate**	**Intrapartum stillbirth rate**
		3. Neonatal mortality rate	Low birth weight rate
			**Preterm birth rate** **Small for gestational age ** **Neonatal morbidity rates** **Disability after neonatal conditions**
Contact point definitions are clear, but data on the content of care is lacking	COVERAGECare for all mothers and newborns	4. Skilled attendant at birth 5. Early postnatal care for mothers & babies 6. Essential newborn care (early breastfeeding)	Antenatal care Exclusive breastfeeding to six months
Gaps in definitions requiring validation and feasibility testing for HMIS use	COVERAGE Complications and extra care	**7. Neonatal resuscitation ** **8. Kangaroo mother care**	Caesarean section rate
		**9. Treatment of serious neonatal infections** **10. Antenatal corticosteroid use**	**Chlorhexidine cord cleansing**
	INPUT Service Readiness for Quality of Care	Emergency obstetric care	
		**Care of small and sick newborns** **Quality of care with measurable norms and standards**	

Bold: Indicators that are not routinely tracked globally

Adapted from *Every Newborn* Action Plan. WHO, 2014. www.everynewborn.org and Mason et al. Lancet 2014

Monitoring progress and ensuring accountability in Low- and Middle-Income Countries has often depended on data from nationally representative household surveys conducted every three to five years, such as TDHS or Multiple Indicator Cluster Surveys (MICS) [22]. These surveys mainly focus on the national and regional levels and are often underpowered by local-level variation, e.g., district. In addition, survey questions lack sufficient granularity, particularly when addressing clinical indicators [27]. Moreover, running costs and infrequent data collection limit their usefulness for tracking rapid changes or emerging health issues.

Routine Health Information Systems (RHIS) are increasingly recognised as crucial data sources for shaping national and subnational strategies and policies [28–30]. The introduction of District Health Information Systems 2 (DHIS2), advancements in digital technology, and easier integration and linkage to web-based systems [7, 31, 32] have provided more significant opportunities for data access and availability for data analysis to monitor trends by facility-level against set targets and global standards [33]. The Tanzania DHIS2 includes newborn indicators reported at the facility level, offering a unique opportunity for analysis focusing on the availability and quality of ENAP indicators. Beyond assessing the availability of these indicators, their practical use for tracking progress, identifying gaps, and informing policy decisions requires continuous evaluation of their quality, including completeness and internal and external consistency.

We are unaware of any study explicitly conducted on DHIS2 routine ENAP indicator data for Tanzania. This paper is part of a supplement reporting findings and learnings from NEST360, an alliance of partners, including four African governments (Kenya, Malawi, Nigeria, and Tanzania), working to reduce neonatal inpatient deaths by improving level-2 newborn care [10] for newborns that require specialised medical attention beyond basic newborn care in hospitals through device installation, training, and quality improvement.

This paper aims to evaluate ENAP core and additional indicator data reported in DHIS2 Tanzania from 2015–2022. The specific objectives are:

1. Assess availability and review numerators, denominators, and definitions of ENAP indicators.
2. Evaluate the completeness of ENAP indicator reporting monthly, both overall and according to health facility levels.
3. Examine the internal consistency of specific ENAP indicator data reported over time.
4. Evaluate the plausibility of reported ENAP indicators compared to Tanzania DHS estimates.

## Methods

### Study setting

**Fig. 1:**
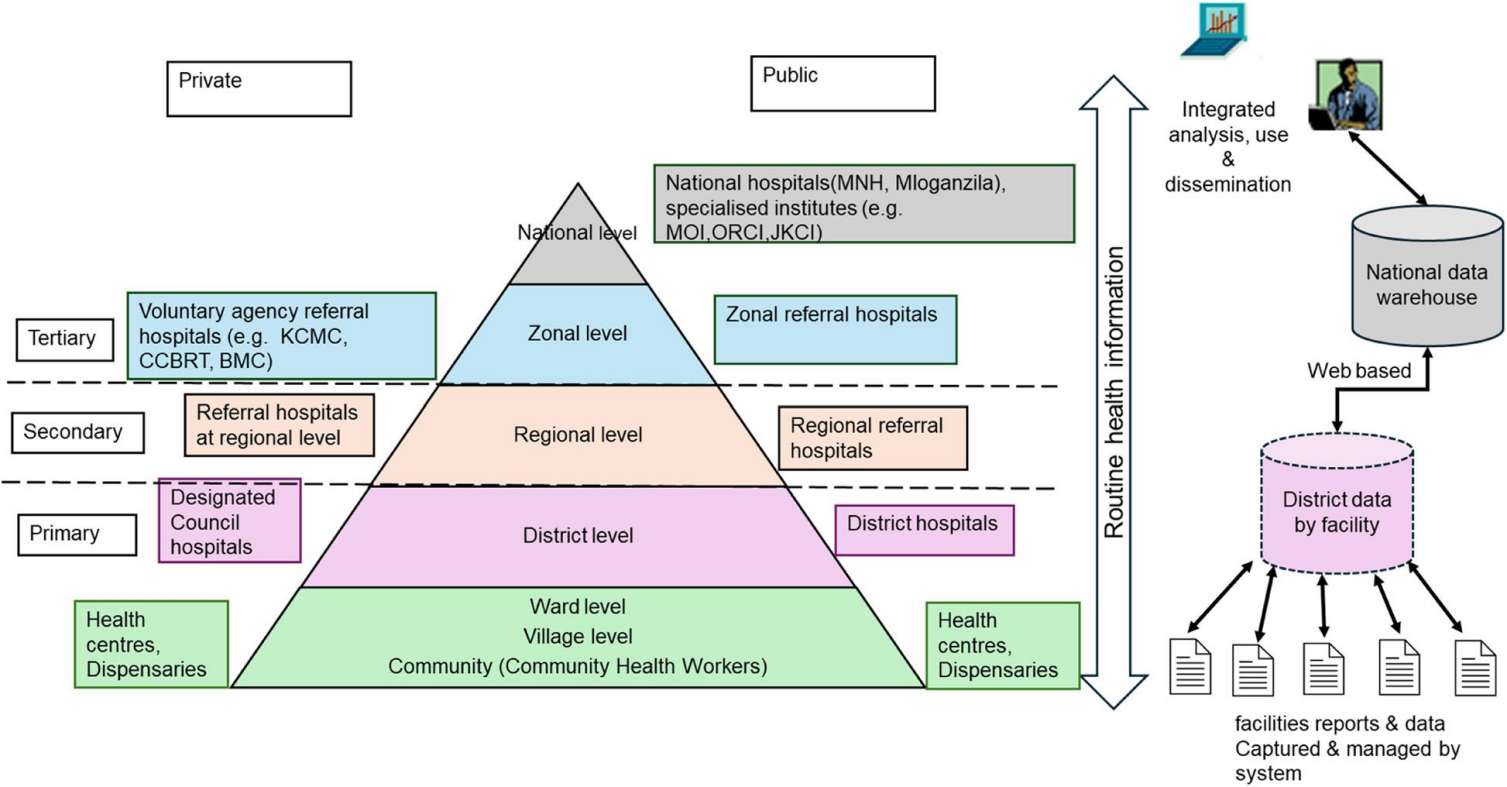
Health system and process of data collation in District Health Information System 2 Tanzania. Abbreviations: KCMC; Kilimanjaro Christian Medical Centre, CCBRT; Comprehensive Community Based Rehabilitation in Tanzania, BMC; Bugando Medical Centre, MNH; Muhimbili National Hospital, MOI; Muhimbili Orthopaedic Institute, ORCI; Ocean Road Cancer Institute, JKCI; Jakaya Kikwete Cardiac Institute

Tanzania, with a population of 61 million in 2022, has been classified as a lower-middle-income country since 2020 [34]. The country has 26 administrative regions, 184 districts, divisions, wards, and villages/streets [34]. The health system in Tanzania is primarily organised as a district health system with a strong emphasis on primary healthcare. The structure of the health system is based on a three-tier system:

- Level 1 (primary) includes i) community services - provided by community health workers(CHWs) and are responsible for delivering essential health education and promotion and some curative services directly within the community, ii) dispensary - is the lowest level typically serving one or a few villages or a ward and primarily focuses on providing out-patient care, iii) health centre - offer a higher level of care compared to the dispensary and serves a larger population is also required to provide inpatient care, often receiving referrals from nearest dispensaries, and iv) district hospital or a designated district hospital located at a district council - serves as the primary referral within the district and provides more comprehensive healthcare services.
- Level 2 (secondary) includes regional referral hospitals, which provide specialist medical care and serve as referral centres for patients requiring advanced medical services beyond the capabilities of primary healthcare facilities.
- Level 3 (tertiary) includes zonal and national hospitals, which offer advanced medical care and serve as teaching hospitals for medical, paramedical, and nursing training. These facilities provide specialised services and are equipped to handle advanced medical care [30, 34] (Fig. 1).

### Data source

The study is based on a secondary analysis of routine health facility data available through the DHIS2 database from 2015–2022. Since 2013, Tanzania’s Health Management Information System (HMIS) has been operating as a hybrid digitalised system. In this system, the initial recording of events is conducted using ward-based paper registers at the facility level, where healthcare workers document primary data from various wards. Subsequently, the data is aggregated using tally and summary sheets before entering into DHIS2 [33, 35]. The monthly paper-based summary forms collected at the health facilities are submitted at the district level. The district information system focal person is primarily responsible for collating all reports and entering data into the DHIS2 system, where data are summarised and aggregated into district, regional, zonal and national levels (Fig. 1). Health centres, district hospitals and tertiary hospitals with HMIS departments do not send paper report forms to the district offices; instead, the data is entered directly into DHIS2 within the health facilities. Some dispensaries submit their monthly reports to the district for entry into the DHIS2 system due to the absence of a dedicated DHIS2 focal person. However, other dispensaries with access to the DHIS2 system upload their data directly from their facilities. Faith-based and private facilities are also expected to submit to the DHIS2 system.

The ENAP indicators are captured in various health facility registers, including labour and delivery, postnatal care, KMC, and death. Currently, neonatal deaths are tallied in the labour ward. However, most deaths are expected to occur and be reported in neonatal care units (NCUs), but unfortunately, official neonatal registers are not yet available.

### Data quality dimensions and analysis

The assessment of ENAP indicator data quality utilised monthly newborn data records from facilities and were analysed according to four quality dimensions adapted from the WHO data quality assessment framework: (1) availability of indicator data, (2) completeness of reporting, (3) internal consistency (4) plausibility by comparing to Tanzania population-based survey estimates(DHS) [36]. Additional File 1 provides further details of these dimensions. We conducted a descriptive analysis using the Stata version18 (StataCorp LLC, Texas, USA).

### Analysis by objectives

#### Objective 1: assess availability and review numerators, denominators, and definitions of indicators

A comprehensive mapping exercise was carried out using the EN-MINI Tool 0 [37] to assess the availability of the ENAP core and additional indicators within DHIS2. The exercise involved mapping the source forms for each indicator, including those related to labour and delivery and postnatal care, to track data flow into the DHIS2 system. The process also involved checking for consistency and validating variables such as birthweight (low or normal), singleton or twin, and type of stillbirth (macerated or fresh) to ensure accurate denominators. This approach enabled the determination of numerators, denominators, and indicator definitions, which were then compared to WHO-Mother and Newborn Information for Tracking Outcomes and Results (MoNITOR) or national guidelines (Table 2). This analysis focused on 12 intrapartum, maternal and newborn care indicators.

**Table 2:** WHO or nationally recommended definitions and availability of ENAP core and additional indicators in national DHIS2, Tanzania

Indicator	Numerator	Denominator	Source document	Available and study focus	Standardised indicator definition
Institutional maternal mortality ratio (per 100,000 live births)	Maternal death in health facilities	Total live births^a^	Labour and delivery register /death registry	✓	✓
The stillbirth rate in a health facility (per 1000 total births)	Number (MSB and FSB) twins & singletons)	Total (live and stillborn) births^a^	Labour and delivery register	✓	✓
Institutional neonatal mortality rate (per 1000 live births)	Number of deaths in health facilities	Total live births^a^	Death registry	✓	✓
Skilled attendant at birth	Number of women assisted by skilled birth attendant	Women (Total): delivered at Health facility + BBA+ Home deliveries + TBA	Labour and delivery register	✓	✓
Postnatal care for newborns	Number of newborns who received postnatal health checks in the first 2 days after birth	Total number of live births^a^	Postnatal care register	✓	✓
Early initiation of breastfeeding	Number of newborns breastfed within one hour of birth	Total livebirths^a^	Labour and delivery	✓	✓
Antenatal corticosteroid use	Not available	Not available	Not available		
Newborn resuscitation with bag and mask	Several newborns were helped to breathe with a bag and mask	Total livebirths^a^	Labour and delivery	✓	✓
Kangaroo Mother Care (KMC)	Number of newborns receiving KMC	Total number of live births^a^ under 2500g	Postnatal care register	✓	✓
Treatment of severe neonatal infections	Number of newborns with septicemia	Total live births^a^	Postnatal care register	✓	✓
Intrapartum stillbirth rate	Number of MSB (Twins + Singletons)	Total (live and stillborn) births^a^	Labour and delivery register	✓	✓
Low birth weight among livebirths	Number of live births^a^ <2.5kg	Total live births^a^	Labour and delivery register	✓	✓
Preterm births^a^	Not available	Not available	Not available		
Small for gestational age	Not available	Not available	Not available		
Neonatal morbidity rates	Not available	Not available	Not available		
Disability after neonatal conditions	Not available	Not available	Not available		
Antenatal care (ANC)+4 visits	Number of ANC 4+ visits	WRA expected to be pregnant	Antenatal care register	x	✓
Exclusive breastfeeding for up to 6 months	Number of infants < 6 months exclusively fed with breast milk	Number of babies under 6 months of age	Child health register	x	✓
Caesarean section rate	Number of caesarean Section deliveries	The sum of modes of deliveries^b^	Labour and delivery register	✓	✓
Chlorhexidine cord cleansing	Not available	Not available	Not available		

Key: ✓; indicator available & focus of the study, x; indicator not focus of the study

*Abbreviations*: *BBA *Born Before Arrival, *TBA *Traditional Birth Attendant, *MSB *Macerated Still Births, *FSB *Fresh Still Births, *WRA *Women of Reproductive Age

^a^Twins + singletons

^b^(breech delivery + caesarean section + spontaneous vaginal deliveries +vacuum deliveries)

#### Objective 2: evaluate the completeness of ENAP indicators reporting every month, overall and by health facility

The completeness of ENAP indicator data was assessed in two stages. First, overall monthly reporting was analysed and evaluated facility-level reporting from 2015–2022. This involved calculating the proportion of missing ENAP indicator data reported monthly at national and facility levels. In DHIS2 Tanzania, entries recorded as “zero” are replaced with empty cells to address server lags and performance issues caused by zeros. However, this approach presents a significant limitation, making it difficult to differentiate between actual missing values (i.e., no data reported) and ‘zero’ values (i.e., no events recorded) since both appear as blank entries. To assess the monthly completeness of ENAP indicators, we assumed all blank entries were missing (i.e. no data reported).

We excluded dispensaries from the analysis of indicator completeness for maternal and newborn outcomes and complications for specific indicators that do not apply to dispensaries, i.e., neonatal resuscitation, kangaroo mother care, treatment of severe neonatal infections, caesarean sections, and mortality outcomes.

Analysis of subnational reporting rates at the regional level was done using DHIS2 data from the labour and delivery and postnatal care reports forms for the period 2015–2022. The percentage of health facilities that offered delivery services and consistently reported data across all 12 months of a year was calculated.

#### Objective 3: examine the internal consistency of specific indicator data reported over time, such as total births and stillbirths

Internal consistency was assessed by (i) the Overall trend of the reported indicator data over time, (ii) Comparing alignment between specific related indicator data, a) Total births and the combined count of live births and stillbirths, b) Stillbirths and the combined count of fresh stillbirths and macerated stillbirths. Individual facilities report stillbirths, live births, and fresh and macerated stillbirths separately. We calculated total stillbirths by summing macerated and fresh stillbirths; we derived live births from combining twins and singleton babies. Total births include live births and stillbirths. Human factors, data entry errors, or inconsistencies in reporting practices can lead to potential discrepancies during the manual addition process. We chose these variables for internal consistency assessment due to their vulnerability to errors and mistakes.

#### Objective 4: plausibility comparison of DHIS2 indicators compared to Tanzania DHS

In Tanzania, facility delivery coverage is more than 80%. To compare with Tanzania DHS, it is essential to consider incomplete reporting in DHIS2 due to home births [12]. We made comparisons for stillbirth rates (SBR), neonatal mortality rates (NMR) and maternal mortality ratio (MMR). The 2022 DHIS2 estimates for MMR, NMR and SBR were 57.1 per 100,000 live births, 5.4 per 1,000 live births and 8.4 per 1,000 births, respectively. We compared these figures with the 2022/23 Tanzania DHS estimates, which reported 104 per 100,000 live births for MMR, 24 per 1000 live births for NMR, and 18.3 per 1000 births for SBR.

## Results

In Tanzania’s mainland, health services are provided by both public and non-public health facilities. By 2020, there were 8,458 health facilities comprising 369 hospitals (including two national hospitals, five zonal referral hospitals, and 28 regional referral hospitals), 926 health centres, and 7,163 dispensaries. Health facilities have steadily increased by (18.9%) from 7,113 in 2015 to 8,458 in 2020 [38, 39]. Since the rollout of DHIS2 in Tanzania in 2015, there has been a nationwide increase in the number of facilities actively reporting to the system—currently, 98% of facilities report data [40]. The distribution of facilities reported in DHIS2 indicates that dispensaries accounted for 65.8%, health laboratories for 12.2%, health centres for 10.1%, clinics for 7.9%, and hospitals for 3.9%. Among these, hospitals exhibited the highest reporting rate, with 92.9% submitting data to DHIS2. This was followed by health centres at 81.2% and dispensaries at 76.7%.

**Fig. 2:**
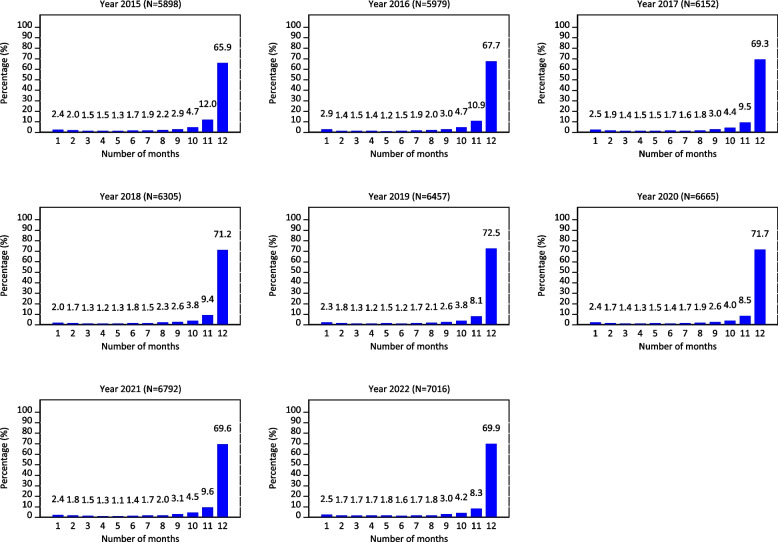
Distribution of facilities offering delivery services by number of reporting months, DHIS2 (2015–2022) data, Tanzania

This improvement is also evident in the reported number of institutional deliveries, which increased by 31.5% from 1,334,150 in 2015 to 1,947,800 in 2022. By 2022, the coverage of public health deliveries in Tanzania reached 80.2%. The proportion of facilities consistently reporting delivery data for all 12 months of the year in DHIS2 increased by 18.9%, rising from 5,898 in 2015 to 7,016 in 2022 (Fig. 2).

### Results by objective

#### Objective 1: assess availability and review numerators, denominators, and definitions of indicators

Tanzania’s DHIS2 platform, which serves as the central health data collection and management system, currently includes 9 out of 10 core ENAP indicators and 5 out of 10 additional ENAP indicators for monitoring. This means that 70% of the indicators specified by ENAP (14 out of 20) are trackable within Tanzania’s DHIS2, with their definitions including their numerators and denominators aligned with WHO-MoNITOR standards or national guidelines. However, determining the total number of live births in DHIS2 requires manual aggregation of singletons and twin counts. ENAP indicators that are not currently included in DHIS2 are antenatal corticosteroid use, preterm births, small for gestational age, neonatal morbidity rates, disability after neonatal conditions and chlorhexidine cord cleansing (Table 2).

#### Objective 2: evaluate the completeness of indicator reporting every month, overall and by health facility

The analysis of ENAP indicator data reporting revealed that > 75% of facilities reported on institutional live births, skilled birth attendance, and early initiation of breastfeeding compared to < 54% that reported on maternal mortality, stillbirths, neonatal mortality, bag and mask ventilation, kangaroo mother care, neonatal infections, low birth weight, and caesarean section) (Table 3).

**Table 3:** Overall summary of the data quality assessment for ENAP indicators in DHIS2, 2015–2022

	Metric		2015	2016	2017	2018	2019	2020	2021	2022
1	Completeness of monthly facility reporting forms	Labor and delivery	98.7	99.4	100.0	100.0	100.0	100.0	90.1	91.4
		Postnatal care	98.3	100.0	100.0	100.0	100.0	100.0	90.5	91.4
2	Percentage of completeness of indicator data reporting (*facilities which reported at least one delivery in the year)*	Institutional livebirths	94.6	95.3	95.7	96.5	97.0	96.7	95.3	96.2
		Institutional maternal Mortality	3.6	3.7	3.9	4.2	4.5	4.8	6.5	6.3
		Stillbirths	31.6	32.8	32.5	32.3	31.4	31.5	29.7	28.0
		Institutional neonatal mortality	21.6	21.7	24.9	22.7	22.8	24.5	29.5	30.4
		Skilled attendant at birth	86.9	87.5	89.1	91.2	93.1	92.7	91.8	92.7
		Postnatal care for newborns	79.9	85.3	88.2	89.8	76.7	91.8	91.2	92.3
		Early initiation of breastfeeding	91.0	92.7	93.2	94.6	95.1	94.8	91.9	93.1
		Newborn resuscitation with bag and mask	28.5	29.8	33.0	34.5	35.0	36.1	34.5	33.6
		Kangaroo mother care	21.2	22.2	25.5	27.0	24.9	30.0	29.6	30.5
		Treatment of severe neonatal infections	3.6	3.8	4.5	3.9	3.7	3.9	3.9	3.8
		Low birth weight among livebirths	45.9	47.4	50.6	52.0	54.7	55.7	55.3	54.7
		Caesarean section	29.3	32.3	35.3	39.0	26.7	49.5	52.5	54.1
3﻿	Percentage of facilities with monthly consistency between stillbirths, live births and total births; *Total births=Stillbirths + Livebirths *	Monthly consistency assessed at 5- standard deviations cutoff	83.6	85.2	85.4	86.4	86.9	87.2	85.9	87.4
4	Percentage of facilities with monthly consistency between Stillbirths, FSB and MSB; *Stillbirths = FSB + MSB*		83.6	85.2	85.4	86.4	86.9	87.2	85.9	87.4

*Abbreviations*: *FSB *Fresh stillbirths, *MSB *Macerated stillbirths

The analysis of indicator data reporting at the facility level revealed notable variation between different facility levels and improvements over time. Health centres experienced the most notable growth in delivery coverage, with a 12.8% increase during this period, while hospital-level deliveries saw an 18.4% decline (Table 4). The percentage of hospitals reporting maternal deaths ranged between 12*–*18%, while < 5% of health centres reported maternal deaths. More than 50% of hospitals reported stillbirths compared to < 22% of health centres. Additionally, over 50% of hospitals reported neonatal deaths, and < 20% of health centres reported neonatal deaths (Table 4). Among the 12 ENAP indicators analysed, hospitals reported a median of eight indicators (interquartile range (IQR) 6*–*9), followed by health centres reporting a median of five indicators (IQR 4*–*6), and dispensaries reporting a median of four indicators (IQR 4*–*4). The annual completeness rate of indicators reporting from the labour, delivery, and postnatal care forms was around 90% (Table 3). Regional analysis of reporting rates revealed that before 2021, 12 out of 26 regions (46%) reported completeness rates slightly exceeding 100%. Furthermore, there has been consistently high yearly reporting completeness at the regional level for both labour and delivery and postnatal care forms. Notable regions with high completeness rates include Dar-es-salaam, Arusha, and Geita (Additional File 2a-2d).

**Table 4:** Percentage of facilities reporting on ENAP indicator data, DHIS2 Tanzania (2015–2022)

Core and additional indicators	Dispensary								Health Centre								Hospital							
	2015	2016	2017	2018	2019	2020	2021	2022	2015	2016	2017	2018	2019	2020	2021	2022	2015	2016	2017	2018	2019	2020	2021	2022
Number of facilities	4674	4819	4956	5094	5215	5356	5453	5621	811	831	855	867	888	921	942	961	261	277	285	288	297	315	337	370
Distribution of institutional deliveries	32.8	32.3	33.4	39.1	40.2	41.3	38.6	38.3	21.4	22.6	24.4	25.4	26.6	29.8	33.1	34.2	45.9	45.2	42.2	35.5	33.2	28.9	28.3	27.5
Institutional livebirths	94.3	95.2	95.5	96.4	96.9	96.5	94.9	95.9	95.0	96.0	96.7	97.3	97.5	97.4	96.9	97.3	95.8	96.8	96.2	97.4	97.4	97.3	96.5	96.5
Maternal mortality	NA	NA	NA	NA	NA	NA	NA	NA	0.8	0.9	1.0	1.4	1.8	2.1	3.5	3.0	11.8	11.9	12.4	12.4	12.4	12.5	18.4	15.2
Stillbirths	NA	NA	NA	NA	NA	NA	NA	NA	18.8	20.7	20.6	21.0	20.5	22.2	20.8	19.3	69.0	68.0	66.6	64.9	62.9	58.7	54.4	50.9
Neonatal mortality	NA	NA	NA	NA	NA	NA	NA	NA	12.2	13.5	15.0	13.1	12.9	14.6	19.5	19.0	49.0	45.6	53.3	50.5	51.4	53.3	58.5	60.7
Skilled attendant at birth	89.3	86.5	88.4	90.7	93.0	92.7	91.5	92.4	90.6	91.7	92.6	92.4	93.3	92.6	93.0	94.1	91.4	92.3	91.6	93.2	93.6	93.3	92.9	93.3
Early postnatal care	80.0	85.5	88.2	89.9	76.8	92.2	91.6	93.0	82.4	86.5	89.7	90.8	77.2	91.5	90.4	91.5	73.2	80.8	84.9	86.5	73.4	87.7	87.0	87.2
Early initiation of breastfeeding	90.5	92.3	92.8	94.3	95.2	94.6	91.4	92.7	92.8	94.4	94.8	95.3	95.7	95.8	94.0	95.3	93.8	95.1	94.9	96.4	95.9	95.4	93.5	94.0
Bag and mask ventilation	NA	NA	NA	NA	NA	NA	NA	NA	16.2	18.4	21.9	24.6	25.6	27.7	27.1	26.3	64.4	62.9	64.8	63.3	62.2	60.4	55.0	53.2
Kangaroo Mother Care	NA	NA	NA	NA	NA	NA	NA	NA	17.6	17.3	20.0	22.0	20.7	25.3	25.3	25.5	31.7	36.5	41.3	41.4	37.2	43.6	41.4	44.0
Neonatal infections	NA	NA	NA	NA	NA	NA	NA	NA	2.6	2.4	2.7	2.3	2.3	2.6	2.3	2.3	6.8	8.0	9.5	8.4	7.8	8.0	8.4	7.6
Low birth weight	NA	NA	NA	NA	NA	NA	NA	NA	34.4	36.3	40.2	42.1	45.8	47.5	47.6	47.1	79.6	79.5	80.5	80.5	80.7	79.7	76.9	74.9
Caesarean section	NA	NA	NA	NA	NA	NA	NA	NA	9.8	13.1	16.3	20.9	26.7	35.6	40.0	42.2	86.2	87.8	90.0	91.3	92.0	90.3	87.2	85.8

#### Objective 3: examine the internal consistency of specific indicator data reported over time, such as total births and stillbirths

Over the eight years, there was a consistently high percentage of facilities reporting institutional live births (> 94%) and early initiation of breastfeeding (> 91%) (Table 3). However, the proportion of facilities reporting treating severe neonatal infections was < 4% for 2015–2022. The percentage of facilities reporting institutional neonatal mortality increased from 21.6 to 30.4%. Additionally, there was a significant increase of over 80% in the proportion of facilities reporting caesarean sections, rising from 24.3% in 2015 to 54.1% in 2022 (Table 3).

When analysing monthly reported data on total stillbirths and their disaggregation into fresh and macerated stillbirths over time, > 84% of facilities demonstrated consistent reporting. However, 13% to 16% of health facilities showed discrepancies in their monthly reported figures for total births compared to the sum of live births and total stillbirths (Table 3).

####  Objective 4: plausibility comparison of DHIS2 indicators compared to Tanzania DHS

The DHIS2 2022 estimates for maternal mortality ratio (MMR), neonatal mortality rate (NMR), and stillbirth rate (SBR) were 57.1 per 100,000 live births, 5.4 per 1,000 live births, and 8.4 per 1,000 births, respectively compared to 104 per 100,000 livebirths, 24 per 1000 livebirths, and 18.3 per 1000 births from the 2022/23 Tanzania DHS. After adjusting for 20% home births, the estimates were 68 per 100,000 live births for MMR, 6 per 1,000 live births for NMR, and 10 per 1,000 births for SBR.

## Discussion

Routine health facility data play a vital role in monitoring service delivery, particularly for tracking the care of small and sick newborns in regions with high neonatal mortality rates, such as Tanzania. This monitoring is crucial, especially as Tanzania is currently off track to achieve sustainable development goal (SDG) 3.2. Our study findings indicate that approximately 70% of ENAP indicators are available within Tanzania’s DHIS2. This level of data availability reflects a moderate to high rate of reporting and recording newborn health indicators within the national health information system. Similar trends have been documented in other studies, underscoring the critical role of routine facility data in monitoring key maternal and newborn health indicators through facility-based data sources [31, 41, 42]. Several factors influence the availability of ENAP indicator data in DHIS2, including the design and accessibility of data collection tools such as registers, well-structured data flow processes, and adequate infrastructure and technology. These components are essential for improving the accuracy and completeness of data reporting, which are critical for effective health information system monitoring and planning [43–46].

In our study, over 90% of health facilities consistently reported ENAP coverage indicators for mothers and newborns, including institutional live births, skilled birth attendance, and early breastfeeding initiation. This indicates that these indicators are systematically monitored and reported over time. However, significant gaps were identified in reporting neonatal mortality, low birth weight, stillbirths, maternal mortality, bag and mask ventilation, treatment of severe neonatal infections, and KMC, particularly at the health centre level. Higher-level facilities may experience both benefits and challenges in reporting and data recording. While the availability of resources and infrastructure can enhance data recording, factors such as high workloads, excessive data demands, and fragmented health information systems can compromise data quality [47–50].

The reporting rates at subnational levels for forms used to collect maternal and newborn data on labour, delivery, and postnatal care are commendable. However, some regions reported percentages exceeding 100%. This anomaly is likely due to certain facilities not being officially documented in the administrative records, newly established facilities or those with inconsistencies in their names, which may lead to inflated reporting rates [31, 33]. Additionally, discrepancies between 13% and 16% were observed between the monthly reported totals for births compared to the combined totals of live births and stillbirths, as well as between total stillbirths and the sum of fresh and macerated stillbirths. These inconsistencies may be attributed to human errors during the manual data aggregation when tallying the reports [33, 51, 52].

Even after a 20% adjustment to account for home births, DHIS2 estimates for MMR, NMR and SBR remain substantially lower than those from Tanzania DHS. In 2022, DHIS2 reported MMR and SBR estimates of 68 and 10, respectively, compared to the 2022/23 Tanzania DHS estimates of 104 and 18.3. Similarly, the NMR reported in DHIS2 was 6, 75% lower than the DHS estimate of 24. It is essential to recognise that neither source can be considered a definitive truth, as both are subject to various biases [53–55]. Institutional reporting of both NMR and MMR can be affected by the tallying and summarisation processes within facilities and by community-level reporting limitations due to the lack of mechanisms for linking community deaths. Furthermore, a significant number of neonatal deaths are likely missed during the data collection in NCUs, which are generally newer compared to maternity and paediatric wards in many facilities [55]. This issue is further exacerbated by the absence of dedicated neonatal registers, which hinders comprehensive and accurate data recording.

The Maternal and Perinatal Death Surveillance and Response (MPDSR) system is also utilised to report maternal mortality in Tanzania. However, it is not integrated with DHIS2 [56, 57]. Integrating these systems could significantly enhance data collection and reporting accuracy and completeness. The underreporting of stillbirth may be linked to the use of differing criteria and thresholds, such as gestational age and/or birthweight [2, 58, 59]. This underscores the need for improved documentation and tallying processes in labour wards.

Additionally, the inadequate recognition of stillbirths in global health metrics highlights the importance of accurate data capture and reporting. Addressing these data gaps is crucial for fully understanding the impact of stillbirths on women and families and for designing effective interventions [4]. The absence of several critical indicators from DHIS2, such as antenatal corticosteroid use, preterm births, small for gestational age, and neonatal morbidity rates, is a significant concern. These indicators are vital for monitoring neonatal health outcomes and ensuring comprehensive health system performance.

### Implications for routine data systems

- Development of a standardised newborn register.

As Tanzania continues to strengthen and expand small and sick newborn care services, it is essential to establish a dedicated inpatient register for capturing key newborn indicators that will enable effective monitoring of outcomes [10, 60]. Integrating the newborn register with DHIS2 will help to streamline data collection and improve data availability.

(b) Shift to individual-level data collection.

The transition from using aggregate data to capturing individual-level data within DHIS2 represents a significant step forward in improving the granularity and utility of health information, particularly for monitoring neonatal health outcomes. Individual-level data capture, such as the NEST360 neonatal inpatient data, allows more precise tracking of indicators for targeted action and interventions. Further, considerations for developing a system that links the mother and baby data within the context of small and sick newborn care are crucial and should be considered [61, 62].

(c) Prioritisation of indicators for inclusion as aggregate DHIS2 indicators.

Including too many indicators in DHIS2 can overwhelm the system, resulting in inefficiencies in data entry, analysis and reporting. It is essential to carefully evaluate and prioritise which newborn ENAP indicators to include, focusing on those that align with national and international priorities and address existing gaps.

(d) Improve data quality and use.

Improving data quality and utilisation is critical for strengthening health information systems, mainly when significant data gaps exist. High-quality data is essential for accurate monitoring, tracking, evaluation, and decision-making to inform effective health policies and interventions.

(e) Harmonisation of digital health systems.

There is a clear need to harmonise and integrate the more than 160 digital health-related systems currently used in Tanzania. Enabling interoperability among various health information systems will enhance the effectiveness and efficiency of healthcare service delivery and lead to better data capture (availability) and monitoring [63].

(f) Review of DHIS2 data validation.

Revisions to DHIS2 validation for Tanzania should prioritise a mechanism for differentiating non-reporting (i.e., no data reported) from “zero” entries, which represent an actual numerical value [64].

### Strengths and limitations

The study spans eight years and encompasses a substantial dataset of over 13 million institutional births, enabling a comprehensive data quality evaluation. Furthermore, DHIS2 is a widely recognised health management information system, offering significant potential for broader scalability.

A significant limitation was the inability to differentiate between actual missing values (i.e. no data reported) and ‘zero’ values (i.e. no events recorded). A similar challenge with zero reporting was observed while assessing the completeness of malaria indicator data reporting through the DHIS2 system in Kenya [65].

### Future research in Tanzania

With over 80% of births occurring in health facilities in Tanzania, there is a significant opportunity to monitor ENAP indicators. Future implementation research at the facility level should focus on identifying barriers to effective data collection and developing context-specific interventions, particularly in lower-level health facilities where most births occur.

## Conclusion

Our study highlights the critical need to strengthen routine facility-based data collection to track maternal and newborn outcomes effectively. Achieving this requires a strong focus on data quality, particularly in addressing the over 75% underreporting of neonatal mortality. While Tanzania has set ambitious goals aligned with ENAP and SDG targets, a comprehensive strategy is essential to ensure reliable data for accurate progress tracking and to build confidence in the quality of DHIS2 data.

## Supplementary Information

Additional file 1. Data quality dimensions adapted from the WHO data quality framework.

Additional file 2. Reporting rate- labor and delivery and postnatal care form (2015-2022)

Additional file 3. Ethical approvals

## Data Availability

The primary data source is the routine health information system. Health facilities report data to district offices every month using standardised reporting forms. Data is entered into computers at the district offices using District Health Information System software version 2 (DHIS2) https://dhis2.org. In the DHIS2, data can be summarised and aggregated at district, regional, zonal, and national levels.

## Abbreviations

CSR: Caesarean Section Rate
DHIS2: District Health Information System 2
ENAP: Every Newborn Action Plan
HMIS: Health Management Information System
KMC: Kangaroo Mother Care
MMR: Maternal Mortality Ratio
NEST360: Newborn Essential Solutions and Technologies
NMR: Neonatal Mortality Rate
PNC: Postnatal Care
SBR: Stillbirth Rate
SDGs: Sustainable Development Goals
TDHS: Tanzania Demographic and Health Survey
WHO: World Health Organization
