# Supplementary material for: Neonatal indicator data in Tanzania District Health Information System: evaluation of availability and quality of selected newborn indicators, 2015-2022
Source: BMC Pediatr. 2025 Jan 23;23(Suppl 2):658. doi: 10.1186/s12887-025-05417-x (PMC11755859; doi:10.1186/s12887-025-05417-x)
Supplement: Supplementary file 1 — Additional file 1. Data quality dimensions adapted from the WHO data quality framework. [file 12887_2025_5417_MOESM1_ESM.docx]

**Additional file 1: Data quality dimensions adapted from WHO data quality framework**

| Dimension | Data quality metric and objective | Level of assessment | Data element/indicator data used | Duration | Analysis |
| --- | --- | --- | --- | --- | --- |
| Availability of indicator data | To assess and map the availability of ENAP indicator data, numerators, denominators definitions in DHIS2 as per recommended WHO-or nationally definitions | National level | Institutional maternal mortality ratio, Stillbirth rate in a health facility, Institutional neonatal mortality, skilled attendant at birth, early postnatal care for babies, early initiation of breastfeeding, newborn resuscitation with bag and mask, kangaroo mother care, treatment of severe neonatal infections, intrapartum stillbirths, low birth weight, caesarean section rate | DHIS2 data; 2015-2022 | Map the available indicator data in DHIS2, numerators and denominators definitions |
| Completeness of indicator data | *(a)Completeness rate of reporting for administrative unit at* *district /reginal level;* | National level and regional level | Monthly reporting forms from labor and delivery and postnatal care | DHIS2 data; 2015-2022 | Completeness rate at national and regional level |
|  | *(b)Completeness of* *indicator data reporting;*  Measures proportion of non-zeros ENAP indicator data reported on monthly basis | National level and facility level | Institutional maternal mortality, Stillbirths, institutional neonatal mortality, skilled attendant at birth, early postnatal care for babies, early initiation of breastfeeding, newborn resuscitation with bag and mask, kangaroo mother care, treatment of severe neonatal infections, intrapartum stillbirths, low birth weight, caesarean section | DHIS2 data; 2015-2022 | Percentage of monthly indicator data values that are non-zero |
| Internal consistency of reported data | *Consistency over time;*  Examines the plausibility between selected related indicator data reported;   1. *Births and the sum of livebirths and stillbirths* 2. *Stillbirths and the sum of fresh and macerated stillbirths* | Facility level  National level | Total births, livebirths, stillbirths, fresh macerated stillbirths, neonatal mortality, caesarean section, maternal mortality | 2015-2022 | Monthly consistency assessed at 5- standard deviations cutoff |
|  |  |  |  |  | Graph plots to visualize the consistency of reported estimates for MMR and SBR; NMR and SBR; MMR and CSR over time |
|  | 1. *Maternal mortality ratio (MMR) and still birth rate (SBR)* 2. *Neonatal mortality rate (NMR) and still birth rate (SBR)* 3. *Maternal mortality ratio (MMR) and caesarean section rate (CSR)* |  |  |  |  |
| External compatibility and plausibility | Examine the level of agreement between estimates for Institutional deliveries, caesarean section, early initiation of breastfeeding, early postnatal care using DHIS2 data and Tanzania Demographic and Health Survey (TDHS) data | Regional and country level | DHIS2 estimates: Institutional deliveries, caesarean section, early initiation of breastfeeding, early postnatal care | 2016, 2022 | Compare and plot the regional estimates (26 regions in Tanzania mainland) |
|  |  |  | TDHS estimates: Institutional deliveries, caesarean section, early initiation of breastfeeding, early postnatal care | 2015/16, 2022/23 |  |
