# Supplementary material for: Neonatal indicator data in Tanzania District Health Information System: evaluation of availability and quality of selected newborn indicators, 2015-2022
Source: BMC Pediatr. 2025 Jan 23;23(Suppl 2):658. doi: 10.1186/s12887-025-05417-x (PMC11755859; doi:10.1186/s12887-025-05417-x)
Supplement: Supplementary file 2 — Additional file 2. Reporting rate- labor and delivery and postnatal care form (2015-2022) [file 12887_2025_5417_MOESM2_ESM.docx]

**Additional file 2a: Reporting rate- labor and delivery form (2015-2018)**

|  | 2015 | | | 2016 | | | 2017 | | | 2018 | | |
| --- | --- | --- | --- | --- | --- | --- | --- | --- | --- | --- | --- | --- |
| Organisation unit | Expected reports | Actual reports | Reporting rate | Expected reports | Actual reports received | Reporting rate | Expected reports | Actual reports | Reporting rate | Expected reports | Actual reports | Reporting rate |
| MOH - Tanzania | 66600 | 65725 | 98.7 | 69000 | 68583 | 99.4 | 70500 | 70586 | **100.1** | 72636 | 73433 | **101.1** |
| Arusha Region | 2496 | 2636 | **105.6** | 2640 | 2707 | **102.5** | 2724 | 2778 | **102.0** | 2856 | 3064 | **107.3** |
| Dar Es Salaam Region | 1320 | 1523 | **115.4** | 1356 | 1852 | **136.6** | 1392 | 1778 | **127.7** | 1476 | 1906 | **129.1** |
| Dodoma Region | 3864 | 3658 | 94.7 | 3996 | 3793 | 94.9 | 4020 | 3866 | 96.2 | 4092 | 3993 | 97.6 |
| Geita Region | 1380 | 1388 | **100.6** | 1476 | 1479 | **100.2** | 1512 | 1548 | **102.4** | 1596 | 1636 | **102.5** |
| Iringa Region | 2520 | 2429 | 96.4 | 2604 | 2535 | 97.4 | 2640 | 2587 | 98.0 | 2712 | 2686 | 99.0 |
| Kagera Region | 3180 | 3248 | **102.1** | 3324 | 3278 | 98.6 | 3336 | 3294 | 98.7 | 3360 | 3315 | 98.7 |
| Katavi Region | 732 | 766 | **104.6** | 792 | 770 | 97.2 | 816 | 751 | 92.0 | 828 | 843 | **101.8** |
| Kigoma Region | 2796 | 2635 | 94.2 | 2808 | 2796 | 99.6 | 2868 | 2813 | 98.1 | 2916 | 2880 | 98.8 |
| Kilimanjaro Region | 3180 | 3008 | 94.6 | 3192 | 3140 | 98.4 | 3228 | 3207 | 99.3 | 3240 | 3287 | **101.5** |
| Lindi Region | 2628 | 2517 | 95.8 | 2640 | 2619 | 99.2 | 2688 | 2698 | **100.4** | 2760 | 2740 | 99.3 |
| Manyara Region | 1908 | 1870 | 98.0 | 1992 | 1926 | 96.7 | 2028 | 1950 | 96.2 | 2160 | 2043 | 94.6 |
| Mara Region | 2688 | 2722 | **101.3** | 2760 | 2902 | **105.1** | 2784 | 2931 | **105.3** | 2940 | 3015 | **102.6** |
| Mbeya Region | 2904 | 2901 | 99.9 | 3084 | 3008 | 97.5 | 3192 | 3238 | **101.4** | 3312 | 3307 | 99.8 |
| Morogoro Region | 3468 | 3373 | 97.3 | 3504 | 3425 | 97.7 | 3576 | 3556 | 99.4 | 3672 | 3695 | **100.6** |
| Mtwara Region | 2232 | 2280 | **102.2** | 2304 | 2333 | **101.3** | 2352 | 2350 | 99.9 | 2364 | 2395 | **101.3** |
| Mwanza Region | 3420 | 3162 | 92.5 | 3600 | 3490 | 96.9 | 3672 | 3562 | 97.0 | 3792 | 3834 | **101.1** |
| Njombe Region | 2856 | 2696 | 94.4 | 2892 | 2801 | 96.9 | 2916 | 2916 | 100.0 | 3072 | 3034 | 98.8 |
| Pwani Region | 2796 | 2779 | 99.4 | 2988 | 2984 | 99.9 | 3252 | 3136 | 96.4 | 3312 | 3389 | **102.3** |
| Rukwa Region | 2244 | 2293 | **102.2** | 2280 | 2310 | **101.3** | 2328 | 2421 | **104.0** | 2424 | 2472 | **102.0** |
| Ruvuma Region | 3252 | 2856 | 87.8 | 3288 | 2958 | 90.0 | 3324 | 3142 | 94.5 | 3480 | 3328 | 95.6 |
| Shinyanga Region | 1980 | 2034 | **102.7** | 2148 | 2148 | 100.0 | 2208 | 2182 | 98.8 | 2280 | 2271 | 99.6 |
| Simiyu Region | 2148 | 2185 | **101.7** | 2280 | 2252 | 98.8 | 2340 | 2334 | 99.7 | 2376 | 2383 | **100.3** |
| Singida Region | 2316 | 2381 | **102.8** | 2424 | 2415 | 99.6 | 2448 | 2491 | **101.8** | 2496 | 2529 | **101.3** |
| Songwe Region | 1812 | 1547 | 85.4 | 1932 | 1625 | 84.1 | 2016 | 1793 | 88.9 | 2088 | 1928 | 92.3 |
| Tabora Region | 3276 | 3218 | 98.2 | 3396 | 3359 | 98.9 | 3444 | 3443 | 100.0 | 3492 | 3476 | 99.5 |
| Tanga Region | 3204 | 3620 | **113.0** | 3300 | 3678 | **111.5** | 3396 | 3821 | **112.5** | 3540 | 3984 | **112.5** |

Note: **Bold** indicates a reporting rate greater than 100%.

**Additional file 2b: Reporting rate -labor and delivery form (2019-2022)**

|  | 2019 | | | 2020 | | | 2021 | | | 2022 | | |
| --- | --- | --- | --- | --- | --- | --- | --- | --- | --- | --- | --- | --- |
| Organisation unit | Expected reports | Actual reports | Reporting rate | Expected reports | Actual reports | Reporting rate | Expected reports | Actual reports | Reporting rate | Expected reports | Actual reports | Reporting rate |
| MOH - Tanzania | 73992 | 75530 | **102.1** | 76032 | 76721 | **100.9** | 88980 | 80177 | 90.1 | 89988 | 82228 | 91.4 |
| Arusha Region | 2916 | 3163 | **108.5** | 2976 | 3265 | **109.7** | 3864 | 3439 | 89.0 | 3900 | 3527 | 90.4 |
| Dar Es Salaam Region | 1512 | 1957 | **129.4** | 1560 | 1374 | 88.1 | 2436 | 2211 | 90.8 | 2484 | 2343 | 94.3 |
| Dodoma Region | 4152 | 4120 | 99.2 | 4236 | 4242 | **100.1** | 4764 | 4396 | 92.3 | 4812 | 4475 | 93.0 |
| Geita Region | 1680 | 1707 | **101.6** | 1704 | 1764 | **103.5** | 2124 | 1860 | 87.6 | 2244 | 2006 | 89.4 |
| Iringa Region | 2820 | 2737 | 97.1 | 2856 | 2857 | 100.0 | 3324 | 2894 | 87.1 | 3348 | 2948 | 88.1 |
| Kagera Region | 3372 | 3367 | 99.9 | 3408 | 3406 | 99.9 | 3768 | 3472 | 92.1 | 3792 | 3581 | 94.4 |
| Katavi Region | 912 | 923 | **101.2** | 924 | 934 | **101.1** | 1248 | 942 | 75.5 | 1260 | 1011 | 80.2 |
| Kigoma Region | 2928 | 2896 | 98.9 | 2940 | 2936 | 99.9 | 3168 | 3005 | 94.9 | 3180 | 3011 | 94.7 |
| Kilimanjaro Region | 3264 | 3328 | **102.0** | 3264 | 3393 | **104.0** | 3852 | 3418 | 88.7 | 3888 | 3570 | 91.8 |
| Lindi Region | 2808 | 2803 | 99.8 | 2844 | 2843 | 100.0 | 3348 | 2894 | 86.4 | 3384 | 2943 | 87.0 |
| Manyara Region | 2184 | 2074 | 95.0 | 2208 | 2200 | 99.6 | 2604 | 2329 | 89.4 | 2628 | 2446 | 93.1 |
| Mara Region | 3024 | 3080 | **101.9** | 3168 | 3336 | **105.3** | 3708 | 3408 | 91.9 | 3756 | 3407 | 90.7 |
| Mbeya Region | 3360 | 3433 | **102.2** | 3444 | 3491 | **101.4** | 3984 | 3607 | 90.5 | 4032 | 3709 | 92.0 |
| Morogoro Region | 3756 | 3890 | **103.6** | 4020 | 4037 | **100.4** | 4404 | 4082 | 92.7 | 4440 | 4159 | 93.7 |
| Mtwara Region | 2412 | 2425 | **100.5** | 2484 | 2530 | **101.9** | 2808 | 2546 | 90.7 | 2832 | 2643 | 93.3 |
| Mwanza Region | 3888 | 4034 | **103.7** | 3984 | 4131 | **103.7** | 4380 | 4186 | 95.6 | 4476 | 4208 | 94.0 |
| Njombe Region | 3096 | 3144 | **101.6** | 3240 | 3295 | **101.7** | 3744 | 3358 | 89.7 | 3756 | 3485 | 92.8 |
| Pwani Region | 3384 | 3485 | **102.9** | 3540 | 3620 | **102.3** | 4212 | 3830 | 90.9 | 4284 | 3870 | 90.3 |
| Rukwa Region | 2436 | 2494 | **102.4** | 2460 | 2528 | **102.8** | 2700 | 2537 | 94.0 | 2700 | 2586 | 95.8 |
| Ruvuma Region | 3540 | 3440 | 97.2 | 3684 | 3652 | 99.1 | 4104 | 3712 | 90.4 | 4104 | 3832 | 93.4 |
| Shinyanga Region | 2352 | 2378 | **101.1** | 2436 | 2439 | **100.1** | 2868 | 2482 | 86.5 | 2880 | 2592 | 90.0 |
| Simiyu Region | 2376 | 2400 | **101.0** | 2460 | 2479 | **100.8** | 2844 | 2542 | 89.4 | 2856 | 2575 | 90.2 |
| Singida Region | 2532 | 2557 | **101.0** | 2604 | 2603 | 100.0 | 3012 | 2637 | 87.5 | 3024 | 2734 | 90.4 |
| Songwe Region | 2184 | 2072 | 94.9 | 2232 | 2169 | 97.2 | 2580 | 2294 | 88.9 | 2628 | 2383 | 90.7 |
| Tabora Region | 3504 | 3561 | **101.6** | 3672 | 3688 | **100.4** | 4284 | 3797 | 88.6 | 4392 | 3827 | 87.1 |
| Tanga Region | 3600 | 4062 | **112.8** | 3684 | 4174 | **113.3** | 4848 | 4299 | 88.7 | 4908 | 4357 | 88.8 |

Note: **Bold** indicates a reporting rate greater than 100%.

**Additional file 2c: Reporting rate- postnatal care form (2015-2018)**

|  | 2015 | | | 2016 | | | 2017 | | | 2018 | | |
| --- | --- | --- | --- | --- | --- | --- | --- | --- | --- | --- | --- | --- |
| Organisation unit | Actual reports | Expected reports | Reporting rate | Actual reports | Expected reports | Reporting rate | Expected reports | Actual reports | Reporting rate | Expected reports | Actual reports | Reporting rate |
| MOH - Tanzania | 68192 | 69348 | 98.3 | 71979 | 71940 | **100.1** | 73560 | 73738 | **100.2** | 75876 | 76592 | **100.9** |
| Arusha Region | 2784 | 2700 | **103.1** | 2998 | 2892 | **103.7** | 3012 | 3131 | **104.0** | 3156 | 3422 | **108.4** |
| Dar Es Salaam Region | 2271 | 1812 | **125.3** | 2484 | 1896 | **131.0** | 1968 | 2612 | **132.7** | 2112 | 2804 | **132.8** |
| Dodoma Region | 3629 | 3912 | 92.8 | 3831 | 4044 | 94.7 | 4068 | 3930 | 96.6 | 4140 | 4038 | 97.5 |
| Geita Region | 1356 | 1380 | 98.3 | 1438 | 1476 | 97.4 | 1500 | 1525 | **101.7** | 1584 | 1624 | **102.5** |
| Iringa Region | 2511 | 2652 | 94.7 | 2608 | 2736 | 95.3 | 2772 | 2676 | 96.5 | 2856 | 2777 | 97.2 |
| Kagera Region | 3219 | 3168 | **101.6** | 3282 | 3312 | 99.1 | 3324 | 3283 | 98.8 | 3348 | 3313 | 99.0 |
| Katavi Region | 747 | 732 | **102.0** | 762 | 792 | 96.2 | 816 | 735 | 90.1 | 828 | 835 | **100.8** |
| Kigoma Region | 2793 | 2892 | 96.6 | 2891 | 2904 | 99.6 | 2964 | 2918 | 98.4 | 3024 | 2966 | 98.1 |
| Kilimanjaro Region | 3522 | 3672 | 95.9 | 3633 | 3708 | 98.0 | 3756 | 3670 | 97.7 | 3804 | 3765 | 99.0 |
| Lindi Region | 2574 | 2700 | 95.3 | 2619 | 2712 | 96.6 | 2760 | 2717 | 98.4 | 2832 | 2792 | 98.6 |
| Manyara Region | 1807 | 1884 | 95.9 | 1904 | 1968 | 96.7 | 2004 | 1902 | 94.9 | 2136 | 2029 | 95.0 |
| Mara Region | 2838 | 2760 | **102.8** | 2934 | 2832 | **103.6** | 2856 | 2985 | **104.5** | 3012 | 3084 | **102.4** |
| Mbeya Region | 3025 | 3000 | **100.8** | 3138 | 3192 | 98.3 | 3288 | 3358 | **102.1** | 3396 | 3403 | **100.2** |
| Morogoro Region | 3637 | 3792 | 95.9 | 3942 | 3828 | **103.0** | 3912 | 3969 | **101.5** | 4020 | 4078 | **101.4** |
| Mtwara Region | 2400 | 2352 | **102.0** | 2478 | 2436 | **101.7** | 2496 | 2478 | 99.3 | 2508 | 2521 | **100.5** |
| Mwanza Region | 3303 | 3444 | 95.9 | 3819 | 3624 | **105.4** | 3708 | 3781 | **102.0** | 3816 | 3958 | **103.7** |
| Njombe Region | 2667 | 2856 | 93.4 | 2815 | 2892 | 97.3 | 2928 | 2908 | 99.3 | 3096 | 3048 | 98.4 |
| Pwani Region | 2792 | 3012 | 92.7 | 3025 | 3228 | 93.7 | 3480 | 3139 | 90.2 | 3552 | 3395 | 95.6 |
| Rukwa Region | 2296 | 2316 | 99.1 | 2340 | 2352 | 99.5 | 2400 | 2475 | **103.1** | 2496 | 2526 | **101.2** |
| Ruvuma Region | 2930 | 3360 | 87.2 | 3169 | 3408 | 93.0 | 3444 | 3265 | 94.8 | 3612 | 3474 | 96.2 |
| Shinyanga Region | 1982 | 1992 | 99.5 | 2184 | 2160 | **101.1** | 2220 | 2191 | 98.7 | 2304 | 2257 | 98.0 |
| Simiyu Region | 2138 | 2148 | 99.5 | 2327 | 2280 | **102.1** | 2340 | 2371 | **101.3** | 2376 | 2389 | **100.5** |
| Singida Region | 2442 | 2364 | **103.3** | 2517 | 2472 | **101.8** | 2496 | 2540 | **101.8** | 2544 | 2581 | **101.5** |
| Songwe Region | 1535 | 1812 | 84.7 | 1638 | 1932 | 84.8 | 2004 | 1746 | 87.1 | 2076 | 1847 | 89.0 |
| Tabora Region | 3303 | 3312 | 99.7 | 3447 | 3432 | **100.4** | 3480 | 3527 | **101.4** | 3540 | 3548 | **100.2** |
| Tanga Region | 3691 | 3324 | **111.0** | 3756 | 3432 | **109.4** | 3564 | 3906 | **109.6** | 3708 | 4118 | **111.1** |

Note: **Bold** indicates a reporting rate greater than 100%.

**Additional file 2d: Reporting rate- postnatal care form (2019-2022)**

|  | 2019 | | | 2020 | | | 2021 | | | 2022 | | |
| --- | --- | --- | --- | --- | --- | --- | --- | --- | --- | --- | --- | --- |
| Organisation unit | Expected reports | Actual reports | Reporting rate | Expected reports | Actual reports | Reporting rate | Expected reports | Actual reports | Reporting rate | Expected reports | Actual reports | Reporting rate |
| MOH - Tanzania | 77280 | 78798 | **102.0** | 79212 | 81400 | **102.8** | 91848 | 83098 | 90.5 | 92808 | 84861 | 91.4 |
| Arusha Region | 3204 | 3555 | **111.0** | 3252 | 3619 | **111.3** | 4068 | 3774 | 92.8 | 4128 | 3881 | 94.0 |
| Dar Es Salaam Region | 2148 | 3021 | **140.6** | 2184 | 3141 | **143.8** | 3312 | 3097 | 93.5 | 3324 | 3127 | 94.1 |
| Dodoma Region | 4200 | 4149 | 98.8 | 4284 | 4311 | **100.6** | 4848 | 4406 | 90.9 | 4896 | 4552 | 93.0 |
| Geita Region | 1668 | 1693 | **101.5** | 1692 | 1749 | **103.4** | 2112 | 1865 | 88.3 | 2232 | 1992 | 89.2 |
| Iringa Region | 2976 | 2835 | 95.3 | 3012 | 2939 | 97.6 | 3456 | 2994 | 86.6 | 3480 | 3082 | 88.6 |
| Kagera Region | 3372 | 3367 | 99.9 | 3408 | 3407 | 100.0 | 3744 | 3468 | 92.6 | 3768 | 3550 | 94.2 |
| Katavi Region | 912 | 915 | **100.3** | 936 | 943 | 100.7 | 1224 | 939 | 76.7 | 1236 | 977 | 79.0 |
| Kigoma Region | 3036 | 2995 | 98.6 | 3048 | 3043 | 99.8 | 3288 | 3117 | 94.8 | 3300 | 3114 | 94.4 |
| Kilimanjaro Region | 3816 | 3823 | **100.2** | 3816 | 3945 | **103.4** | 4176 | 3976 | 95.2 | 4212 | 3949 | 93.8 |
| Lindi Region | 2916 | 2842 | 97.5 | 2952 | 2928 | 99.2 | 3420 | 2982 | 87.2 | 3444 | 3042 | 88.3 |
| Manyara Region | 2160 | 2071 | 95.9 | 2196 | 2193 | 99.9 | 2580 | 2303 | 89.3 | 2604 | 2393 | 91.9 |
| Mara Region | 3096 | 3158 | **102.0** | 3240 | 3389 | **104.6** | 3804 | 3464 | 91.1 | 3852 | 3479 | 90.3 |
| Mbeya Region | 3456 | 3521 | **101.9** | 3540 | 3579 | **101.1** | 4056 | 3691 | 91.0 | 4092 | 3798 | 92.8 |
| Morogoro Region | 4104 | 4205 | **102.5** | 4356 | 4390 | **100.8** | 4884 | 4476 | 91.6 | 4932 | 4574 | 92.7 |
| Mtwara Region | 2556 | 2552 | 99.8 | 2628 | 2682 | **102.1** | 2976 | 2694 | 90.5 | 3000 | 2766 | 92.2 |
| Mwanza Region | 3912 | 4058 | **103.7** | 3960 | 4075 | **102.9** | 4428 | 4063 | 91.8 | 4524 | 4108 | 90.8 |
| Njombe Region | 3120 | 3158 | **101.2** | 3264 | 3315 | **101.6** | 3756 | 3317 | 88.3 | 3768 | 3466 | 92.0 |
| Pwani Region | 3624 | 3473 | 95.8 | 3768 | 3604 | 95.6 | 4224 | 3820 | 90.4 | 4296 | 3857 | 89.8 |
| Rukwa Region | 2508 | 2551 | **101.7** | 2532 | 2587 | **102.2** | 2712 | 2608 | 96.2 | 2712 | 2635 | 97.2 |
| Ruvuma Region | 3672 | 3589 | 97.7 | 3816 | 3780 | 99.1 | 4212 | 3838 | 91.1 | 4212 | 3932 | 93.4 |
| Shinyanga Region | 2376 | 2366 | 99.6 | 2460 | 2445 | 99.4 | 2868 | 2503 | 87.3 | 2868 | 2603 | 90.8 |
| Simiyu Region | 2376 | 2411 | **101.5** | 2472 | 2496 | **101.0** | 2820 | 2543 | 90.2 | 2832 | 2570 | 90.7 |
| Singida Region | 2580 | 2612 | **101.2** | 2652 | 2664 | **100.5** | 3024 | 2698 | 89.2 | 3036 | 2768 | 91.2 |
| Songwe Region | 2184 | 2069 | 94.7 | 2232 | 2177 | 97.5 | 2544 | 2281 | 89.7 | 2592 | 2354 | 90.8 |
| Tabora Region | 3552 | 3597 | **101.3** | 3684 | 3703 | **100.5** | 4332 | 3767 | 87.0 | 4440 | 3828 | 86.2 |
| Tanga Region | 3756 | 4212 | **112.1** | 3828 | 4296 | **112.2** | 4980 | 4414 | 88.6 | 5028 | 4464 | 88.8 |

Note: **Bold** indicates a reporting rate greater than 100%.
